# Case of Strangulated Hernia Reduced En Masse, with Observations

**Published:** 1846-10

**Authors:** Robert Wade

**Affiliations:** Senior Surgeon to the Westminster General Dispensary


*Case of Strangulated Hernia reduced* en masse, *with Observations.* By Robert Wade, Esq., Senior Surgeon to the Westminster General Dispensary.—The patient, a man in his seventy-fifth year, who had been afflicted with inguinal hernia on both sides for nearly thirty years, was seized with the usual symptoms of strangulation. On examination no appearance of hernia could be detected. Purgatives and enemata failed in procuring evacuations. The circumstance of a slight darting pain having been experienced by the patient in the right inguinal region, on getting out of bed on the day that he was taken ill, and that on coughing the hernia descended on the left side, and could not be made to protrude on the right, led to the conclusion, that the right hernia, with its investing sac, had been reduced *en masse* by the patient, and that the obstruction existed in the neck of the sac on that side. The author accordingly operated on the right side, in the evening of the second day. The inguinal canal having been freely laid open by a division of the tendon of the external oblique muscle, the sac, which was much thickened and closely embraced the intestine, was opened. It contained a small knuckle of congested intestine. A membranous band, distant as far as the finger could reach, was divided, and the strangulated intestine was then reduced. The patient afterwards recovered.—*Ibid.*

